# Giant Retinal Pigment Epithelium Tear Secondary to Hypotony After Trabeculectomy for Open-Angle Glaucoma With Preoperative Untreated Choroidal Neovascularization: A Case Report

**DOI:** 10.7759/cureus.103122

**Published:** 2026-02-06

**Authors:** Tsuyoshi Matsuno, Ryo Tomita, Jun Takeuchi, Koji M Nishiguchi, Kenya Yuki

**Affiliations:** 1 Department of Ophthalmology, Nagoya University Graduate School of Medicine, Nagoya, JPN

**Keywords:** choroidal neovascularization, intraocular pressure, primary open angle glaucoma, retinal pigment epithelium tear, serous retinal detachment, trabeculectomy

## Abstract

Retinal pigment epithelium (RPE) tears are frequently observed following anti-vascular endothelial growth factor therapy for age-related macular degeneration. While rare, giant RPE tears have also been reported secondary to choroidal detachment (CD) induced by postoperative hypotony after trabeculectomy (TLE). Although mechanical stress on the RPE is considered a common underlying factor in both scenarios, the exact mechanisms remain unclear. This report describes a case of a giant RPE tear originating from the site of a previously untreated choroidal neovascularization (CNV) following TLE.

A 65-year-old male underwent TLE for intraocular pressure (IOP) control in his left eye with primary open-angle glaucoma. Preoperative IOP was 39 mmHg, and fundus examination revealed untreated CNV and sub-RPE hemorrhage. The scleral flap sutures were sequentially laser lysed on postoperative days two and four, as the postoperative IOP remained stable between 12 and 14 mmHg. On postoperative day seven, IOP decreased to 4 mmHg, and CD was observed. Following observation, the IOP increased to 7 mmHg on postoperative day 10, revealing a giant RPE tear and serous retinal detachment (SRD). An additional scleral flap suture was performed on the same day, and the IOP subsequently stabilized around 10 mmHg. During follow-up, the SRD spontaneously resolved by postoperative day 41, while the RPE tear persisted. Visual field testing revealed worsening of visual field defects compared to preoperative findings, with defects corresponding to the location of the RPE tear. The rapid IOP reduction following TLE may have induced mechanical stress on a vulnerable RPE region affected by CNV, leading to the RPE tear. A rapid IOP reduction may increase the risk of an RPE tear when vulnerable RPE areas exist due to CNV or other factors; therefore, careful preoperative evaluation for vulnerable RPE regions and cautious perioperative IOP management should be considered.

## Introduction

Retinal pigment epithelium (RPE) tears are ruptures of the RPE that can occur in association with age-related macular degeneration (AMD), particularly in eyes with serous pigment epithelial detachment (PED) and/or subretinal fluid (SRF), as well as other conditions that produce PED. These tears are considered to result from mechanical stretching and subsequent rupture of the RPE and may cause sudden, significant visual loss. However, the precise causes remain unclear, leading to uncertainties in treatment strategies [1,2]. Known etiologies of RPE tears include intravitreal injections of anti-vascular endothelial growth factor (anti-VEGF) agents for AMD, polypoidal choroidal vasculopathy (PCV), retinal angiomatous proliferation (RAP), post-retinal detachment surgery, panuveitis, choroidal tumors, and trauma [2,3]. Although less frequently reported, giant RPE tears have also been documented as a complication of glaucoma filtration surgery, including trabeculectomy (TLE), secondary to postoperative hypotony and associated choroidal detachment (CD) [1,4-7].

TLE is often performed in cases where low target intraocular pressure (IOP) is crucial due to its significant IOP-lowering effect. However, the procedure is technically demanding, requiring careful management, including the timing of scleral flap suture lysis. Postoperative hypotony-related complications, such as hypotony maculopathy, ocular decompression retinopathy (ODR) [8], and CD, warrant close attention and meticulous management. While some reports have described a RPE tear occurring early after glaucoma filtration surgery, including TLE, in conjunction with hypotony [4-7], others have documented a giant RPE tear occurring as late as 108 days postoperatively due to prolonged CD associated with hypotony [1], making the timing of RPE tear development unpredictable. To better inform surgical decisions and perioperative IOP management, it is clinically important to identify preoperative fundus findings that may signal RPE vulnerability. However, no previous reports have identified preoperative fundus findings suggestive of vulnerable RPE areas, such as choroidal neovascularization (CNV). This report presents a case where a giant RPE tear developed early postoperatively due to hypotony following TLE, with retrospective identification of preoperative CNV. This case suggests that the RPE tear originated from this area. These findings highlight the potential clinical value of preoperative fundus evaluation to detect vulnerable RPE regions in patients undergoing TLE.

## Case presentation

**Figure 1:**
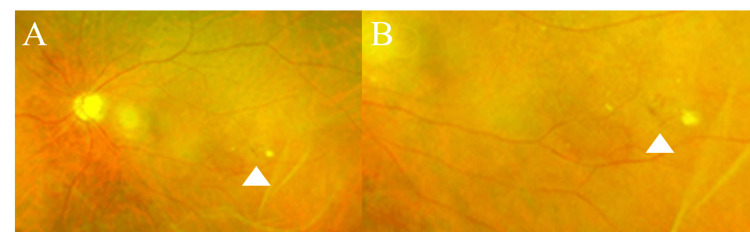
Preoperative fundus photography Spot hemorrhage inferior to the arcade vessels (A, B). The white arrowheads indicate spot hemorrhages.

**Figure 2:**
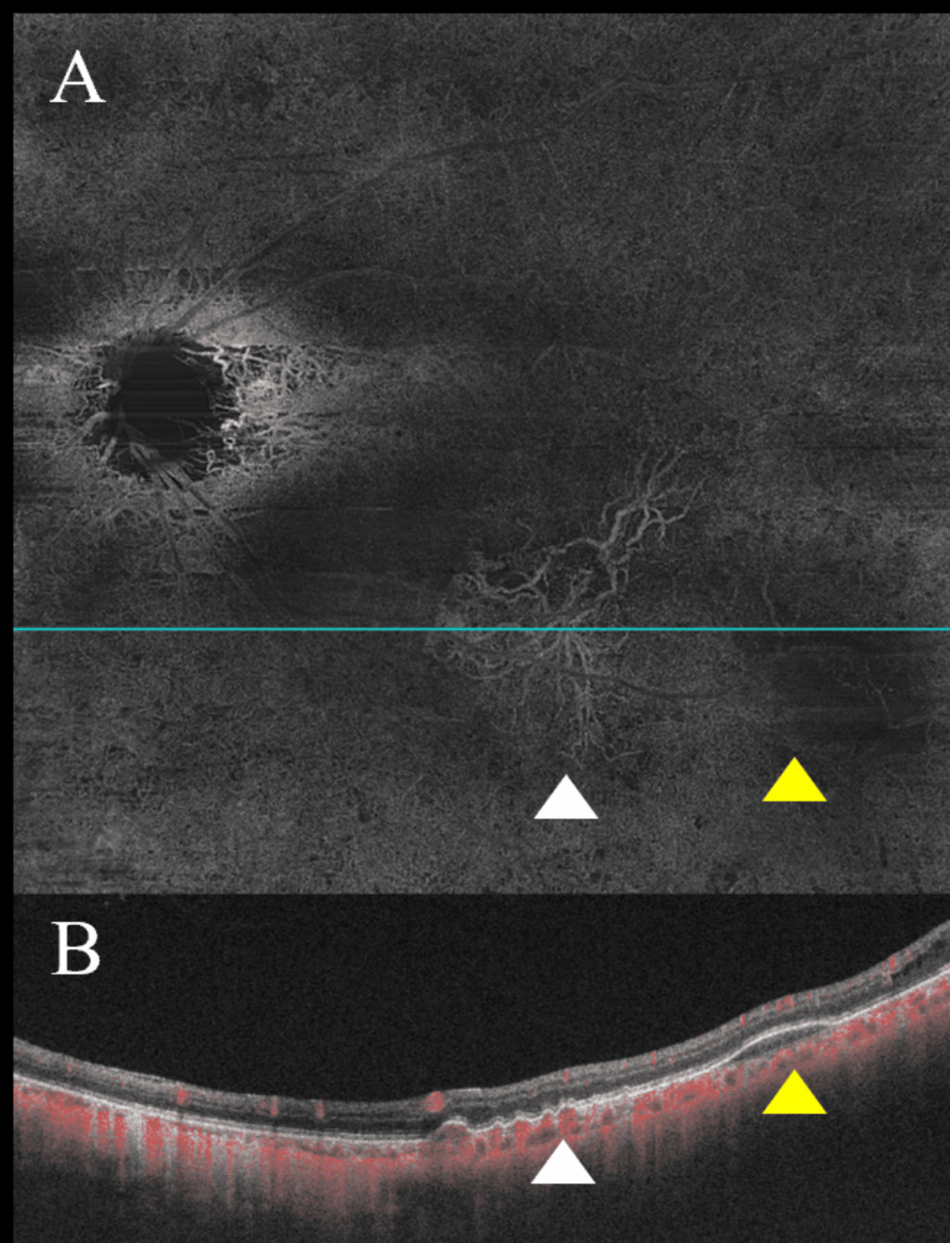
Preoperative optical coherence tomography angiography Choroidal neovascularization (CNV) and sub–retinal pigment epithelium (RPE) hemorrhage on preoperative optical coherence tomography angiography (OCTA) (A, B). (A) shows an en face OCTA image; (B) shows the corresponding B-scan obtained along the blue line indicated in (A). White arrowheads indicate CNV, and yellow arrowheads indicate sub-RPE hemorrhage. Red signals represent blood flow. OCTA was obtained using a swept-source OCTA system (Plex Elite 9000; Carl Zeiss Meditec, Jena, Germany) with a 12 × 12 mm scan. An en face OCTA image was generated using device-based automated segmentation. The CNV was evaluated on an en face OCTA slab from the outer plexiform layer to Bruch’s membrane. Areas showing increased flow signal (displayed in red on the flow overlay) were interpreted as reflecting blood flow.

**Figure 3:**
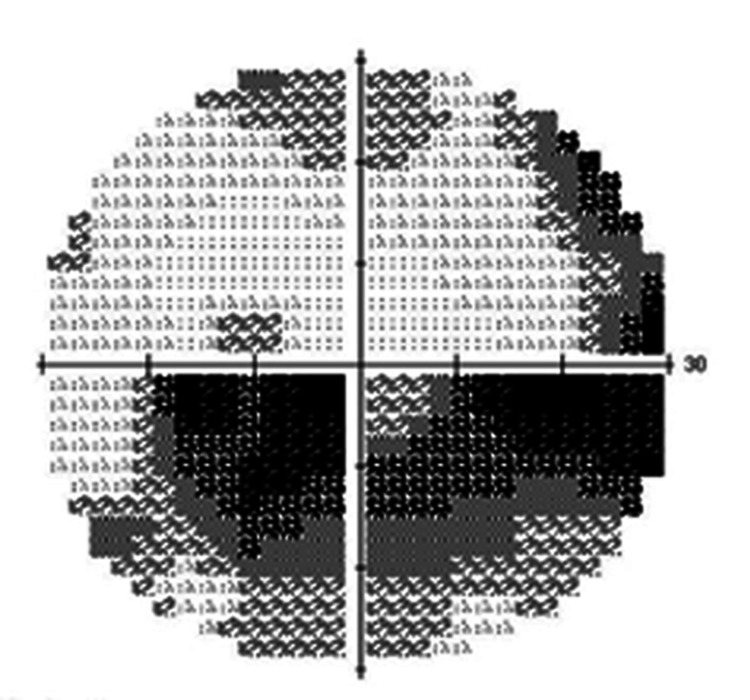
Preoperative Humphrey Field Analyzer 30-2 Visual field examination showed a predominantly inferior visual field defect.

A 65-year-old male with primary open-angle glaucoma in his left eye was referred for surgical management due to uncontrolled IOP despite using five topical glaucoma medications and oral medication. At the initial examination, his best-corrected visual acuity (BCVA) on the logMAR scale was 0 in the right eye and 0.22 in the left eye. His past ocular history included bilateral simple diabetic retinopathy (Figure 1). The right eye was phakic, and the left eye was pseudophakic. IOP was 18 mmHg in the right eye and 39 mmHg in the left eye. Fundus examination revealed optic disc cupping in both eyes, more pronounced with rim thinning in the left eye (Figure 1). CNV and sub-RPE hemorrhage were noted in the left eye (Figure 2), but he had no prior history of anti-VEGF intravitreal injections. At presentation, the CNV was considered inactive (quiescent), with no apparent subretinal or intraretinal fluid; therefore, anti-VEGF treatment was not initiated. Anterior chamber depth was 3.33 mm in the right eye and 5.15 mm in the left eye. Axial length was 26.11 mm in the right eye and 26.37 mm in the left eye. Humphrey Field Analyzer (HFA) showed an inferior visual field defect with central involvement in the left eye (Figure 3). Table 1 summarizes the clinical course, key findings, and interventions over time.

**Table 1:** Clinical course, key findings, and interventions Abbreviations: BCVA, best-corrected visual acuity; CNV, choroidal neovascularization; IRF, intraretinal fluid; IOP, intraocular pressure; MD, mean deviation; NR, not recorded; PED, pigment epithelial detachment; POD, postoperative day; Preop, preoperative; PRN, pro re nata; RPE, retinal pigment epithelium; SRD, serous retinal detachment; SRF, subretinal fluid; VF, visual field; VFI, visual field index; VEGF, vascular endothelial growth factor.

Time point	IOP (mmHg)	BCVA (logMAR)	Key findings (macula/fundus)	Intervention
Preop	39	0.52	CNV with sub-RPE hemorrhage; no SRF/IRF (quiescent)	—
POD2	14	NR	—	Laser suture lysis
POD4	8	NR	—	Laser suture lysis
POD7	4	NR	Choroidal detachment (predominantly temporal)	Observation
POD10	7	NR	PED, RPE tear, SRD	Additional scleral flap suture
POD16	8	NR	Giant RPE tear, SRD	—
POD41	13	NR	SRD resolved; RPE tear persisted	Observation
POD112	12	0.6	VF progression (MD −20.48 dB)	—
POD283	18	1.3	New drusenoid deposits with perilesional PED and new SRF (CNV reactivation)	Anti-VEGF injection
POD476	19	1.22	—	Anti-VEGF injection (PRN)
POD646	18	1.3	—	Anti-VEGF injection (PRN)

**Figure 4:**
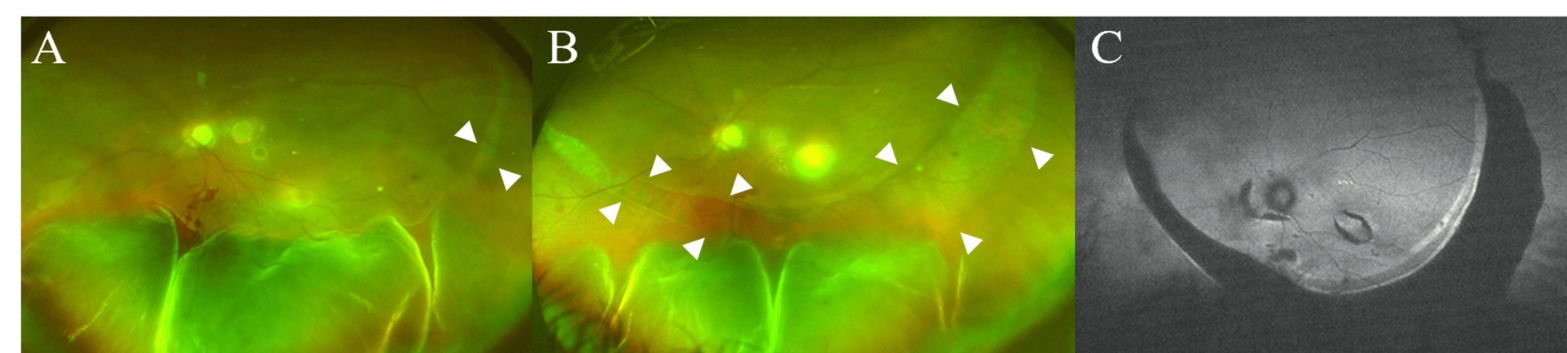
Postoperative fundus photography Fundus photography at postoperative day 10 (A). Fundus photography at postoperative day 16 (B). Fundus autofluorescence at postoperative day 16 (C). The white arrowheads in the fundus photographs indicate retinal pigment epithelium tear.

**Figure 5:**
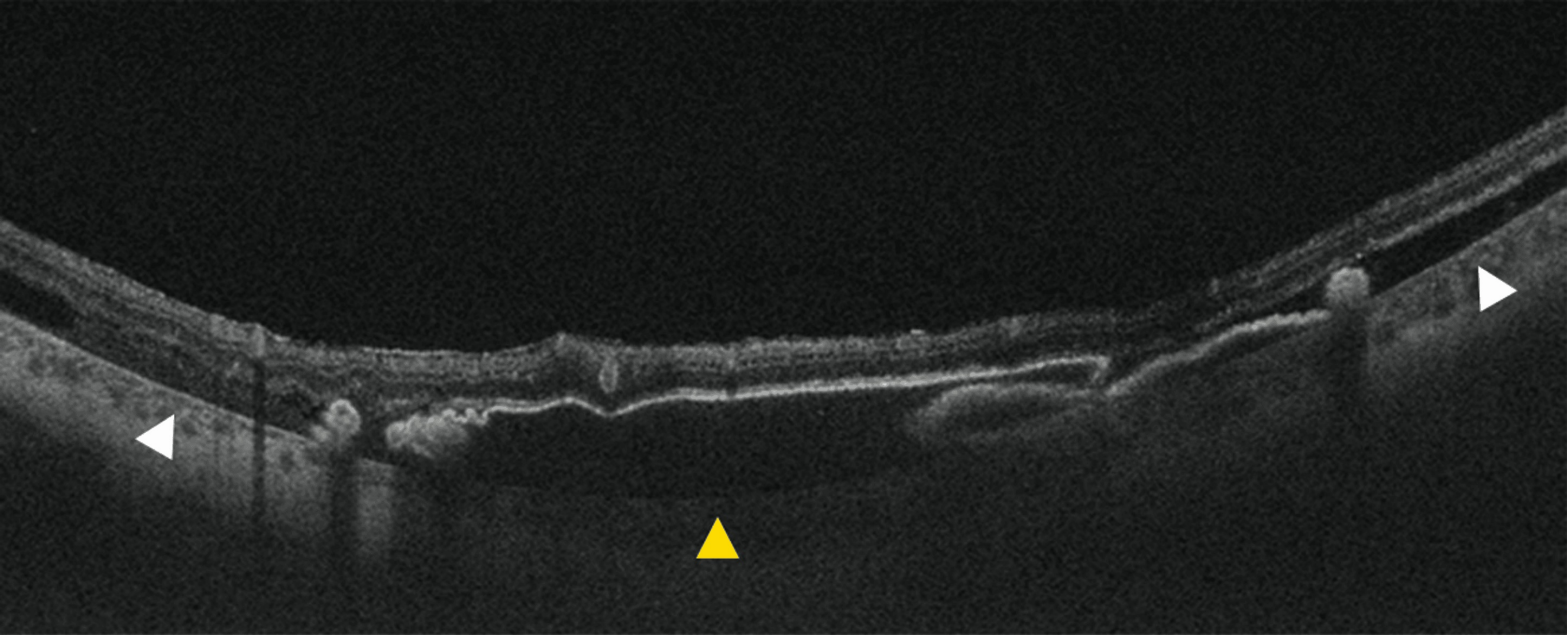
Postoperative optical coherence tomography Optical coherence tomography at postoperative day 16. The white arrowheads indicate retinal pigment epithelium (RPE) tear. The yellow arrowhead indicates retinal RPE detachment.

**Figure 6:**
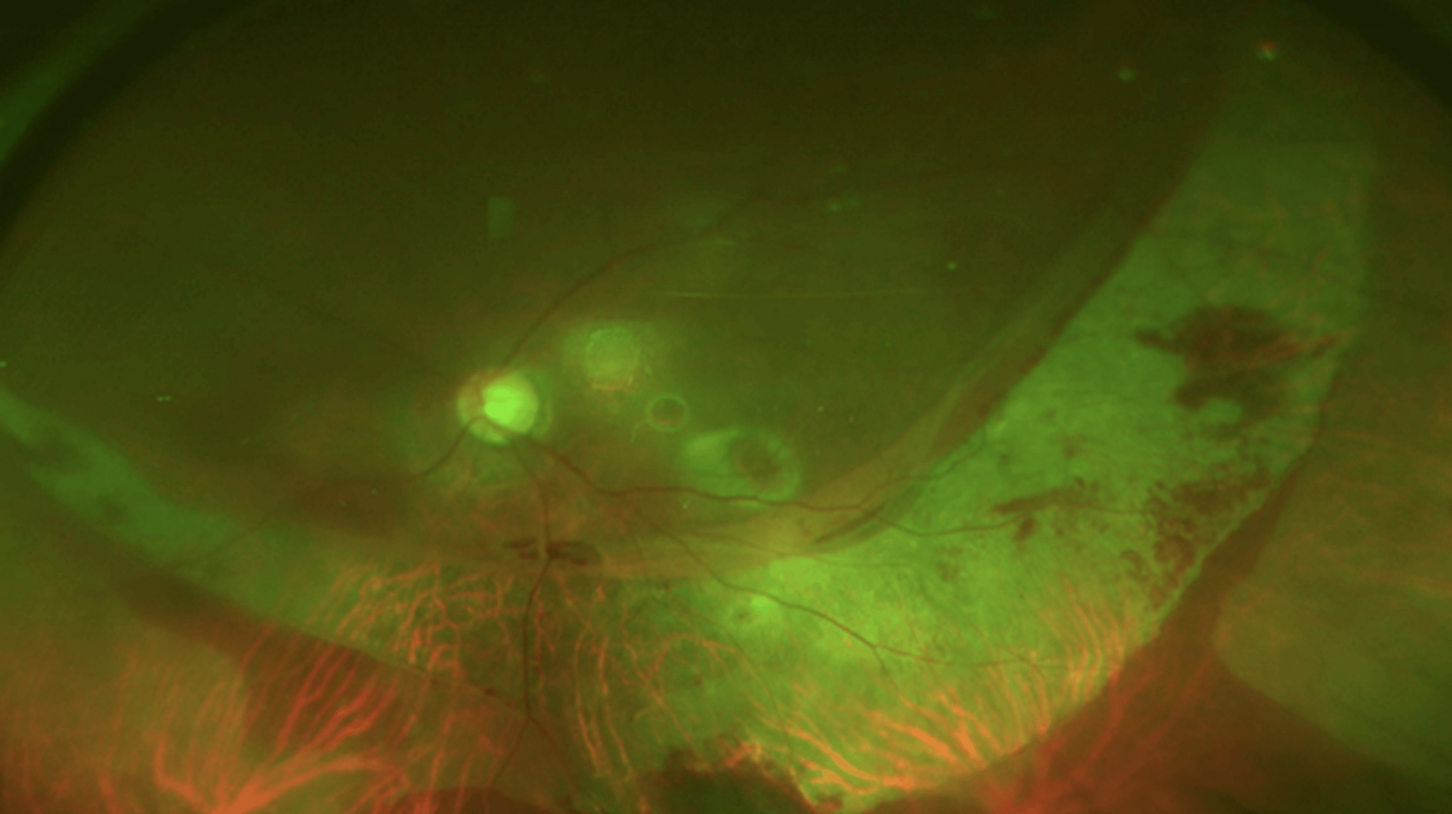
Fundus photography at postoperative day 41 The serous retinal detachment resolved, but a large retinal pigment epithelium tear remained.

**Figure 7:**
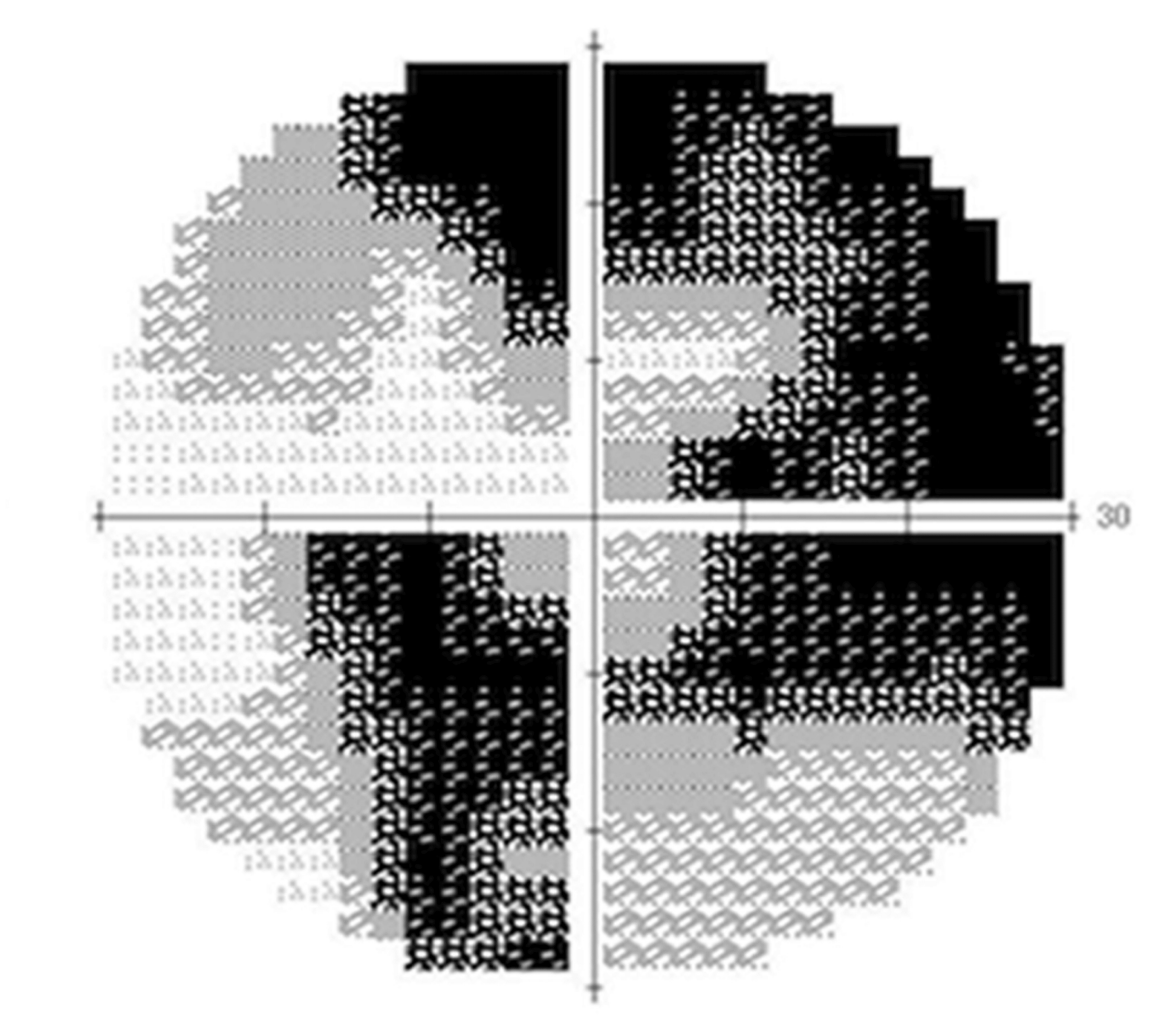
Postoperative Humphrey Field Analyzer 30-2 Compared to preoperative findings, progression of superior visual field defects corresponding to the location of the retinal pigment epithelium tear was observed.

**Figure 8:**
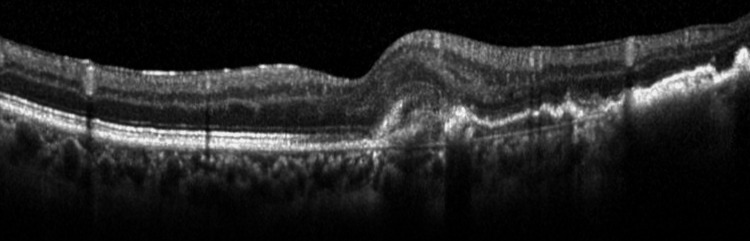
Optical coherence tomography at postoperative day 283 Drusenoid deposits and subretinal fluid were observed in the macular region.

TLE was performed on the left eye for IOP control and was completed without complications. On postoperative day two, IOP was stable at 14 mmHg, and laser suture lysis was performed. On postoperative day four, laser suture lysis was repeated due to an IOP of 12 mmHg. On postoperative day seven, although the anterior chamber remained deep and a functional bleb was observed, IOP decreased to 4 mmHg, and CD was noted on fundus examination, predominantly extensive in the temporal quadrant (imaging not available). On postoperative day 10, IOP was 7 mmHg, and a PED, RPE tear, serous retinal detachment (SRD), and new retinal hemorrhages were observed (Figure 4). An additional scleral flap suture was placed. Subsequently, IOP remained stable around 10 mmHg, and CD showed a tendency to improve. A giant RPE tear and SRD were confirmed on postoperative day 16 (Figure 4). Optical coherence tomography (OCT) showed PED and RPE tear (Figure 5). With observation only, SRD gradually improved and resolved by postoperative day 41, although the giant RPE tear persisted (Figure 6). On postoperative day 112, IOP was well-controlled at 12 mmHg, but BCVA decreased to 0.60 on the logMAR scale, and HFA showed worsening of superior visual field defect (MD: −13.00 dB preoperatively vs −20.48 dB at POD112) (Figure 7), which appeared topographically compatible with the location of the RPE tear. Due to the decreased vision, anti-VEGF intravitreal injection was performed for CNV on postoperative day 283. At that time, macular OCT demonstrated newly developed drusenoid deposits with a perilesional PED and newly developed subretinal fluid, suggestive of CNV reactivation (Figure 8). A pro re nata treatment regimen was adopted, and anti-VEGF intravitreal injections were performed on postoperative days 476 and 646. No further intravitreal injections have been administered since then.

## Discussion

RPE tears have been reported to occur in the macular region following intravitreal injections of anti-VEGF agents for conditions such as AMD, PCV, and RAP [2,3]. They have also been reported in association with CD and SRD secondary to hypotony after TLE, as in the present case [1,4,6]. Although a RPE tear after TLE is rare, factors other than mechanical stretching of the RPE during hypotony may also contribute to triggering their development [1-4,6]. Other causes of RPE tears include post-panretinal photocoagulation, post-panuveitis, and trauma. A common factor among these conditions may be that mechanical stress is applied to the RPE when vulnerable areas are present [2,3]. Therefore, in the development of RPE tears following TLE, the presence of vulnerable RPE areas that could serve as a starting point for the RPE tear, in addition to the occurrence of CD or SRD, may be important. In the present case, previously untreated CNV was identified upon retrospective review of preoperative images, indicating a potentially vulnerable RPE area. The postoperative RPE tear also occurred in an area including the periphery of the CNV, suggesting that it may have been the starting point. To date, no reports of RPE tears occurring after glaucoma filtration surgery have demonstrated the presence of a preoperative vulnerable RPE area due to CNV as in this case. Given the many remaining unknowns regarding the mechanism of RPE tear development, this case is considered useful for elucidating the cause. Furthermore, when performing TLE in cases with vulnerable RPE areas, such as those with CNV, it may be necessary to initiate anti-VEGF therapy beforehand if there is time before surgery, or to control IOP gradually and slowly postoperatively if surgery is urgent, with cautious scleral flap suture lysis to avoid abrupt IOP reduction. The limitation of this case is that it is a report of only one case, and increasing the number of cases is necessary to elucidate the mechanism of RPE tear development and to develop guidelines for managing TLE in patients at risk for vulnerable RPE areas. In this case, based on the imaging-defined localization of the CNV and the RPE tear, we suggest that CNV-associated RPE vulnerability may have predisposed the affected area to an RPE tear following choroidal detachment. However, it remains uncertain whether CNV was necessarily involved in the development of the RPE tear, and the possibility that the tear occurred independently of CNV cannot be excluded. ODR may have been present in the current case. ODR can occur following rapid hypotony after glaucoma filtration surgery, such as TLE, and is primarily characterized by retinal hemorrhages and occasionally subretinal detachment [8]. Although ODR may cause RPE stretching, there are no prior reports of this condition leading to an RPE tear.

## Conclusions

This case suggests that in patients with vulnerable RPE areas, including pre-existing CNV, the development of SRD or CD secondary to hypotony after TLE may trigger RPE tears originating from these vulnerable sites. Accordingly, preoperative screening to identify vulnerable RPE areas and to stabilize CNV, along with avoidance of abrupt perioperative IOP reduction and careful postoperative IOP control, should be considered.
